# Association between obstructive sleep apnea risk and type 2 diabetes among Emirati adults: results from the UAE healthy future study

**DOI:** 10.3389/fendo.2024.1395886

**Published:** 2024-07-16

**Authors:** Manal Taimah, Amar Ahmad, Mohammad Al-Houqani, Abdulla Al Junaibi, Youssef Idaghdour, AbdiShakur Abdulle, Raghib Ali

**Affiliations:** ^1^ Public Health Research Center, New York University Abu Dhabi, Abu Dhabi, United Arab Emirates; ^2^ Department of Medicine, College of Medicine and Health Sciences, UAE University, Al-Ain, United Arab Emirates; ^3^ Department of Pediatrics, Zayed Military Hospital, Abu Dhabi, United Arab Emirates; ^4^ MRC Epidemiology Unit, University of Cambridge, Cambridge, United Kingdom

**Keywords:** obstructive sleep apnea, type 2 diabetes, gender differences, obesity, UAEHFS, United Arab Emirates

## Abstract

**Introduction:**

Obstructive sleep apnea (OSA) can have negative impacts on the health outcomes of individuals with type 2 diabetes. However, in the United Arab Emirates (UAE), there is a lack of understanding regarding the relationship between OSA and type 2 diabetes despite the significant implications it has on health. The primary objective of this study is to investigate the association between OSA risk and type 2 diabetes, associated risk factors, and gender differences in OSA symptoms among Emirati adults.

**Methods:**

We conducted a cross-sectional analysis of the baseline data from the UAE Healthy Future Study (UAEHFS) collected between February 2016 and March 2023. Our sample consisted of 4578 participants aged 18-71 who completed the STOP-BANG survey, provided body measurements and blood samples. We stratified the patients according to their OSA risk and diabetes. We used univariate and multivariate logistic regression models to analyze the relationship between OSA risk and type 2 diabetes and to identify factors associated with risk for OSA and type 2 diabetes. We estimated odds ratios (ORs) with corresponding 95% confidence intervals (95% CI).

**Results:**

The mean age was 27.5 years (± 8.35), and 55.81% (n=2555) were men. The overall prevalence of high risk for OSA was 16.58% and was higher in men compared to women (26.46% vs 4.10%). Women reported feeling tired more often than men (68.02% vs 48.96%). Both genders have similar rates of stop breathing and BMI ≥ 35. There was a significant association between the OSA risk and type 2 diabetes in the unadjusted model (OR=2.44; 95% CI: 1.78-3.35; p-value <0.0001) and (OR=6.44; 95% CI: 4.32-9.59; p-value < 0.0001) among those who reported intermediate and high OSA risk, respectively. After adjusting the model for education attainment, marital status, waist circumference, and smoking, the association remained significant between diabetes and OSA risk, with an OR of 1.65 (95%CI: 1.18-2.32; p-value =0.004) for intermediate OSA risk and 3.44 (95%CI: 2.23-5.33; p-value <0.0001) for high OSA risk.

**Conclusions:**

This study conducted in the UAE found a significant correlation between OSA risk and type 2 diabetes. We suggest introducing routine screening of OSA for individuals with diabetes.

## Introduction

Obstructive sleep apnea (OSA) is an underdiagnosed sleep disorder characterized by a recurrent episode of partial or complete upper-airway collapse causing cessation in ventilation, sleep apnea, hypoxia, and fragmented sleep (1, 2). OSA is a chronic health condition associated with serious cardiovascular and metabolic comorbidities, road traffic accidents, decreased work productivity, and mortality (3–6). The overall prevalence of OSA ranged from 9% to 38% in general population (7). A cross-sectional study conducted in Dubai showed that 21% of participants were at high risk of OSA (8). Another study found that 20.0% of Kuwaiti adults had a high OSA risk (9).

A pathophysiological mechanism suggested that OSA can trigger an inflammatory response in the body, impacting hemostasis and resulting in insulin resistance and cardiometabolic dysfunction (1, 4, 10). A cross-sectional study conducted in Saudia Arabia found that 15.2% of type 2 diabetes patients are at high risk for OSA using the STOP-BANG questionnaire (11). Another cross-sectional study conducted in the United Kingdom found that the prevalence of high OSA risk among type 2 diabetes men was around 57% using the Berline questionnaire (12). Previous studies showed that OSA can worsen glucose outcomes among type 2 diabetes patients and increase the risk for diabetes related complications such as cardiovascular diseases, kidney disease and mortality (6, 13–15).

The risk factors for OSA can be classified as non-modifiable or modifiable factors. Non-modifiable factors include age, sex, race, and genetics (2, 16). Modifiable risk factors can include obesity, alcohol consumption, smoking, and the use of certain medications like muscle relaxants (2, 17, 18). Previous evidence has yielded conflicting results on the relationship between gender and OSA among type 2 diabetes patients (16, 19–21). Some studies showed a higher prevalence of OSA in men with type 2 diabetes (19, 21). However, some other studies have found a higher prevalence of OSA in women with type 2 diabetes (16, 20). In the United Arab Emirates (UAE), there is limited knowledge about the differences in the risk of OSA and type 2 diabetes between males and females.

The UAE is currently facing a significant health challenge due to the increasing prevalence of diabetes and obesity. According to the International Diabetes Federation, the age-adjusted diabetes prevalence rate is currently at 16.4%, and it is expected to rise to 18.1% by 2045 (22). Emirati nationals are more prone to diabetes than other ethnic groups in the UAE, such as non-national Arabs, Asians, Africans, and Westerners (23). The global standard for diabetes prevalence is 6.1%, which is substantially lower than the rate reported in the UAE (16.4%) (22, 24). According to the national health survey, almost 71% of UAE citizens are overweight, with 37% being classified as obese, which makes the UAE one of the countries with the highest obesity rates worldwide (25). Obesity and diabetes are prevalent health problems (22, 25). Therefore, it is important to understand the significance of these conditions and their association with OSA. Previous studies did not provide adequate information on the correlation between OSA and type 2 diabetes, gender differences, reported sleep symptoms, and related risk factors among Emirati adults. Therefore, this study aims to investigate the relationship between OSA and type 2 diabetes among adult Emirati participants, given the elevated rates of obesity and type 2 diabetes in the UAE. Additionally, the study seeks to identify the risk factors that increase the likelihood of OSA in patients with type 2 diabetes and the differences between both sexes. As this is a cross-sectional analysis, the direction of the association cannot be determined.

## Materials and methods

### Study design, setting, and participants

**Figure 1:**
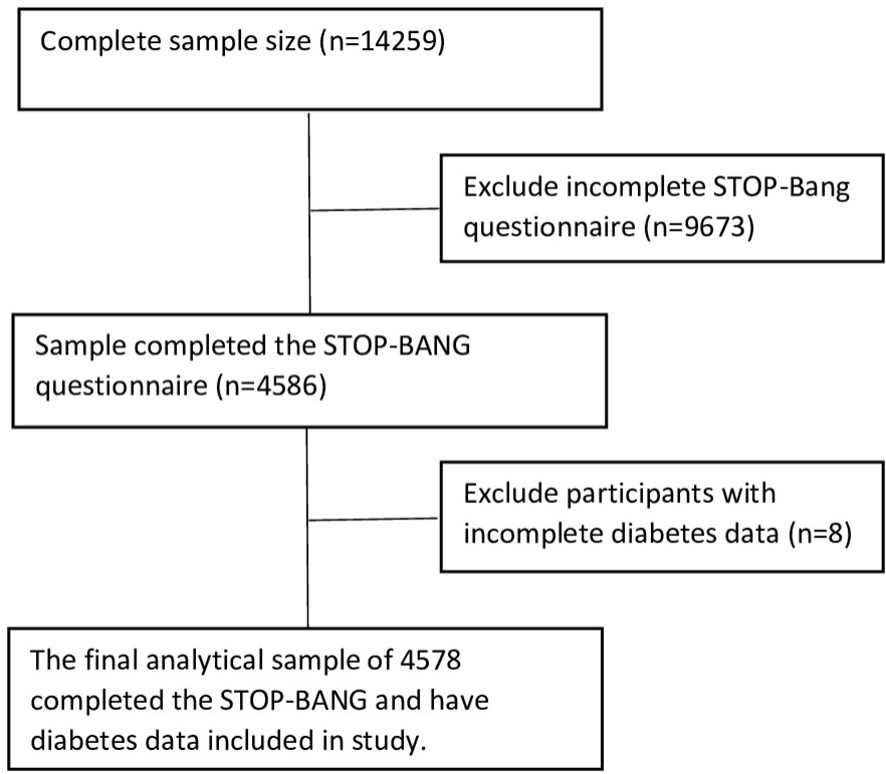
Flow diagram of participant inclusion, exclusion and sample for final Analysis.

This cross-sectional study used baseline data from the United Arab Emirates Healthy Future Study (UAEHFS), collected between February 2016 and March 2023. The UAEHFS is an ongoing longitudinal study that aims to recruit 20,000 Emirati nationals to better understand non-communicable diseases’ causes and risk factors. The UAHFS was conducted across several recruitment centers in Abu Dhabi, Al-Ain, Dubai, Sharjah, and Ras Al Khaimah. Eligible participants were Emirati adults aged 18 years and above who were able to provide consent. Pregnant women and non-Emirati individuals were excluded. Convenience sampling was used to gather information by inviting participants who visited the recruitment centers to complete an online survey in Arabic or English, provide biological samples, and undergo body measurements. The UAEHFS methodology is explained in detail elsewhere (26, 27). We included ***4578 participants*** who completed the STOP-BANG questions and provided body measurements and blood samples (**Figure 1**).

### Ethical approval

The UAEHFS was conducted in adherence to the guidelines established by the Declaration of Helsinki. The study was reviewed and approved by several institutions, including the New York University Abu Dhabi Institutional Review, Dubai Health Authority, Ministry of Health and Prevention in the UAE, and Health Research and Technology Committee. The reference number for the study is DOH/HQD/2020/516. Before data collection, all eligible participants were required to provide written consent after thoroughly reviewing the information leaflet and asking questions. An anonymized electronic dataset, with all personal information removed, was obtained to address the research question of this study.

### Definition of the primary outcome

Type 2 diabetes was the primary outcome of this study. We considered the following criteria for assessing diabetic status: 1) fasting blood glucose of greater than or equal to 126 mg/dl; 2) having a hemoglobin A1C (HbA1C) of greater than or equal to 6.5%; 3) random blood glucose of greater than or equal to 200 mg/dl; 4) self-reported of physician diabetes diagnosis confirmed by being on medication for treating diabetes that was collected in the survey (28). Standard laboratory methods were used to collect and analyze blood samples from participants (26).

### STOP-BANG questionnaire

The STOP-BANG questionnaire is a screening tool designed to assess the risk of OSA. The STOP-BANG questionnaire in Arabic was validated, showing high sensitivity (97.7%) and negative predictive values (92%) in detecting high OSA (Apnea-hypopnea index ≥30) using polysomnography (29). It consists of eight questions related to various health and demographic variables. The survey includes both self-reported and clinical measured parameters divided into two categories: STOP (snoring, tiredness, observed apnea, and high blood pressure) and BANG (body mass index (BMI), age, neck circumference, and gender). Healthcare professionals utilize the STOP-BANG questionnaire to identify individuals who may be at risk of developing OSA (30). The STOP-BANG questionnaire is a widely used tool that has shown high sensitivity in detecting people with low, intermediate, and high OSA risk (89.1%, 90.7%, and 93.9%, respectively) (31).

### OSA risk classification criteria

According to the STOP-BANG criteria, we divided the participants into three categories based on their risk score for OSA: low, intermediate, and high (30). Those who answered “Yes” to two or fewer questions were considered to have a low risk of OSA, while those who answered 3 to 4 “Yes” were considered to have an intermediate risk of OSA. Individuals who answered 5-8 questions with “Yes” or two or more “Yes” responses on the STOP questionnaire, along with one of the following: Male sexes or BMI >35 kg/m2 or neck circumference of 43 cm for males and 41 cm for females were considered at high risk of OSA.

### Biometric measurements

The participant’s age, gender, height, weight, and neck girth were measured to assess the risk of OSA. Participants were asked to fill in their age and gender in the demographic section of the survey, which was verified using their Emirate identification card. Marital status was measured by asking the participants to choose their current status and were classified as Single, married, or other (divorced or widow). The Seca Stadiometer digital scale was used to measure height, a Seca-200 tape was used for neck circumference, and TANITA was used to calculate body composition and BMI. The cut-off values for neck circumference were 43 cm for males and 41 cm for females (30). The cut-off values for average waist circumference were < 80 cm for women and < 94 cm for men (32). The World Health Organization criteria used to classify BMI as: Underweight (BMI < 18.5), average weight (BMI of 18.5-24.9), overweight (BMI of 25.0-29.9), obese class I (BMI of 30-34.9), and obesity class II and III (BMI≥35) (33). Blood pressure (Bp) was measured twice, and the average of the two readings was used for analysis. High blood pressure was defined as meeting any of the following criteria: 1) systolic BP ≥140 mmHg and/or diastolic BP ≥ 90 mmHg; 2) report of physician diagnosed of hypertension; 3) current treatment with BP-lowering drug(s) prescribed for treating high BP (34). Participants were classified as smokers if they have smoked more than 100 cigarettes in their lifetime and if they currently smoke any of the following at least once a month: cigarettes or midwakh/dokha or shisha/waterpipe.

### Statistical analysis

The characteristics of the study participants are presented as mean and standard deviation (SD) for continuous variables or frequency and percentages for categorical and binary variables. Student t-test or ANOVA was used for continuous variables to determine whether there was a statistically significant difference between groups. The degree of skewness was computed for each continuous variable by OSA and diabetes groups to assess the normality assumption. For categorical variables, the chi-squared test was employed. Participants were classified as having or not having diabetes according to the criteria mentioned in the methods section. We utilized univariate and multivariate logistic regression models to analyze the relationship between type 2 diabetes and OSA risk groups adjusted for educational attainment, marital status, waist circumference, and smoking. Odds ratios (ORs) with corresponding 95% confidence interval (95% CI) were estimated. To compare the performance of the univariate model (with OSA as a predictor) and the multivariate logistic regression models, we used Akaike’s information criterion (AIC). For the main analysis, we conducted a complete case analysis by including participants with complete data for the main variables of interest (OSA risk and type 2 diabetes). For covariates with missing values (education and smoking status), we employed a missing indicator method to incorporate all available data and reduce potential bias caused by missing data. All statistical tests were two-sided, and we considered P values <0.05 statistically significant. All statistical analysis was completed using the STATA version 17.0 (35).

To assess the robustness of our findings, we conducted a sensitivity analysis using five multiple imputations and fitted a logistic regression model with diabetes as an outcome and OSA as the main predictors adjusted for waist circumference, marital status, education attainment and smoking. The default number of multiple imputations (m=5) is commonly used and recommended in the literature (36). The results were summarized using Rubin’s rules, providing combined estimates and confidence intervals (37). This approach ensures that our conclusions remain reliable even when accounting for potential missing data (38).

## Results

A total of 4578 adults with a mean age of 27.5 ± 8.35 years, of which 2555 (55.81%) were male and 2023 (44.19%) were female, enrolled in the analysis. Weak to moderate skewness was observed for continuous variables by OSA and diabetes groups. **Table 1** presents the baseline characteristics of the respondents according to OSA risk. Based on the OSA risk criteria, 75.82% of the participants were classified as low OSA risk, 7.60% as intermediate OSA risk, and 16.58% as high OSA risk. Compared to low OSA risk, fewer women had a high OSA risk (4.10% vs 92.29%), while 26.46% of men had high OSA risk compared to 62.78% of men with low OSA risk. Compared to low OSA risk, participants with high OSA risk were older (34.17 ± 9.51 vs 26.25 ± 7.51), had higher BMI (36.74 ± 6.97 vs 24.97 ± 5.15), greater neck circumference (42.39 ± 3.07 vs 34.28 ± 3.60) and wider waist circumference (112.83 ± 15.16 vs 81.40 ± 12.71). A higher proportion of participants with high blood pressure had High OSA risk (50.51%) compared to low and Intermediate OSA risk (34.52% and 14.97%, respectively). More smokers were in the low OSA risk group compared to intermediate and high OSA risk (60.02%, 10.74% and 29.26%).

**Table 1:** General characteristics of the study participants by obstructive sleep apnea risk severity.

Characteristics	Low OSA risk 3471 (75.82%)	Intermediate OSA risk 348 (7.60%)	High OSA risk 759 (16.58%)	P Values
Age, mean (±SD)	26.25 (7.51)	30.97 (9.37)	34.17 (9.51)	<0.0001

Gender, n (%)				<0.0001
Male	1604 (62.78)	275 (10.76)	676 (26.46)	
Female	1867 (92.29)	73 (3.61)	83 (4.10)	

Marital status, n (%)				<0.0001
Single	2,366 (83.11)	158 (5.55)	323 (11.35)	
Married	997 (63.46)	171 (10.88)	403 (25.65)	
Others	108 (67.50)	19 (11.88)	33 (20.62)	

Education attainment, n (%)				<0.0001
Middle school or less	122 (59.22)	30 (14.56)	54 (26.21)	
Secondary school	1514 (76.62)	151 (7.64)	311 (15.74)	
University or more	1726 (76.61)	157 (6.97)	370 (16.42)	
Missing PN, DN	109 (76.22)	10 (6.99)	24 (16.78)	

BMI, mean (±SD)	24.97 (5.15)	29.91 (6.62)	36.74 (6.97)	<0.0001
BMI, n (%)				<0.0001
≤24.9	1882 (90.70)	46 (2.22)	147 (7.08)	
25-29.9	1060 (76.15)	88 (6.32)	244 (17.53)	
30-34.9	404 (59.68)	121 (17.87)	152 (22.45)	
≥35	125 (28.80)	93 (21.43)	216 (49.77)	

Neck circumferences, mean (±SD)	34.28 (3.60)	38.76 (3.67)	42.39 (3.07)	<0.0001
Neck circumference, n (%)				
Below cut-off value	3356 (85.03)	104 (2.63)	487 (12.34)	<0.0001
Above cut-off value	115 (18.23)	244 (38.67)	272 (43.11)	

Waist circumference, mean(±SD)	81.40 (12.71)	96.33 (14.66)	112.83 (15.16)	<0.0001
Waist circumference, n (%)				<0.0001
Below cut-off value	2400 (86.18)	81 (2.91)	304 (10.92)	
Above cut-off value	1071 (59.73)	267 (14.89)	455 (25.38)	

Diabetic status, n (%)				< 0.0001
No	3362 (77.0)	315 (7.21)	689 (15.78)	
Yes	109 (51.42)	33 (15.57)	70 (33.02)	

Having High blood pressure, n (%)				<0.0001
No	3132 (87.10)	201 (5.59)	263 (7.31)	
Yes	339 (34.52)	147 (14.97)	496 (50.51)	

Smoking, n (%)				<0.0001
No	2519 (82.83)	182 (5.98)	340 (11.18)	
Yes	761 (60.02)	136 (10.73)	371 (29.26)	
Missing, PN, DN	191 (71.00)	30 (11.15)	48 (17.84)	

The total sample (n=4578) values are presented as numbers (%) or mean (±SD). Continuous variables by OSA status, were tested using ANOVA, and a Chi-squared test was used for categorical variables. Missing data represents "Prefer not to answer" or "Do not know" or missing. If data was missing, a missing indicator was added to the table. OSA, obstructive sleep apnea; SD, standard deviation; BMI, body mass index; PN, prefer not to answer; DN, do not now.

**Table 2** shows the characteristics of participants by diabetes status. The prevalence of type 2 diabetes in our sample was 4.63%, with 4.70% of females and 4.58% of males having diabetes. Compared to non-diabetic, type 2 diabetes participants were older than non-diabetic (34.93 ± 11.03 vs 27.18 ± 8.03), had higher mean BMI (30.31 ± 6.95 vs 26.31 ± 6.24), larger neck circumference (37.27 ± 4.66 vs 35.45 ± 4.25), and broader waist circumference (96.01 ± 17.62 vs 85.30 ± 15.42). There were more participants with university education or more in the non-diabetic group compared to the diabetes group (95.56% vs 4.44%). In our sample, out of 982 individuals with high blood pressure, 9.67% had type 2 diabetes, while 90.33% did not have diabetes. Compared to the non-diabetic group, smoking was less common among diabetic group (6.07% vs 93.93%).

**Table 2:** General characteristics of the study participants by diabetes status.

Characteristics	No Diabetes 4366 (95.37%)	Diabetes 212 (4.63%)	Total n=4578	P Values
Age, mean (± SD)	*27.18 (8.03)*	*34.93 (11.03)*	*27.54 (8.35)*	*<0.0001*
Gender, n (%)				*0.852*
Male	*2438 (95.42)*	*117 (4.58)*	*2555*	
Female	*1928 (95.30)*	*95 (4.70)*	*2023*	
Marital status, n (%)				*<0.0001*
Single	*2765(97.12)*	*82 (2.88)*	*2847*	
Married	*1454 (92.55)*	*117 (7.45)*	*1571*	
Others	*147 (91.88)*	*13 (8.13)*	*160*	
Education attainment, n (%)				*0.014*
Middle school or less	*187 (90.78)*	*19 (9.22)*	*206*	
Secondary school	*1888 (95.55)*	*88 (4.45)*	*1976*	
University or more	*2153 (95.56)*	*100 (4.44)*	*2253*	
Missing PN, DN	*138 (96.50)*	*5 (3.50)*	*143*	
BMI, mean (± SD)	*26.31 (6.24)*	*30.31 (6.95)*	*26.49 (6.33)*	*0.025*
BMI, n (%)				*<0.0001*
≤24.9	*2030 (97.83)*	*45 (2.17)*	*2075*	
25-29.9	*1332 (95.69)*	*60 (4.31)*	*1392*	
30-34.9	*620 (91.58)*	*57 (8.42)*	*677*	
≥35	*384 (88.48)*	*50 (11.52)*	*434*	
Neck circumference, mean (± SD)	*35.45 (4.25)*	*37.27 (4.66)*	*35.54 (4.29)*	
Neck circumference n (%)				*<0.0001*
Below cut-off value	*3795 (96.15)*	*152 (3.85)*	*3947*	
Above cut-off value	*571 (90.49)*	*60 (9.51)*	*631*	
Waist circumference, mean (± SD)	*85.30 (15.42)*	*96.01 (17.62)*	*85.80 (15.67)*	*0.005*
Waist circumference, n (%)				*<0.0001*
Below cut-off value	*2,714 (97.45)*	*71 (2.55)*	*2785*	
Above cut-off value	*1652 (92.14)*	*141 (7.86)*	*1793*	
OSA status, n (%)				
Low-OSA risk	*3362 (96.86)*	*109 (3.14)*	*3471*	
Intermediate OSA risk	*315 (90.52)*	*33 (9.48)*	*348*	
High OSA risk	*689 (90.78)*	*70 (9.22)*	*759*	
Having High blood pressure, n (%)				*<0.0001*
No	*3479 (96.75)*	*117 (3.25)*	*3596*	
Yes	*887 (90.33)*	*95 (9.67)*	*982*	
Smoking, n (%)				*0.015*
No	*2918 (95.96)*	*123 (4.04)*	*3041*	
Yes	*1191 (93.93)*	*77 (6.07)*	*1268*	
Missing, PN, DN	*257 (95.54)*	*12 (4.46)*	*269*	

The total sample (n=**4578)** values are presented as numbers (%) or mean (± SD).

Continuous variables by diabetes status were tested using the Student t-test. The chi-squared test was used for categorical variables. Missing data represents choices of “Prefer not to answer” or “Do not know” or missing. If data was missing, a missing indicator was added to the table.

SD, standard deviation; BMI, body mass index; PN, prefer not to answer; DN, do not know.

**Figure 2:**
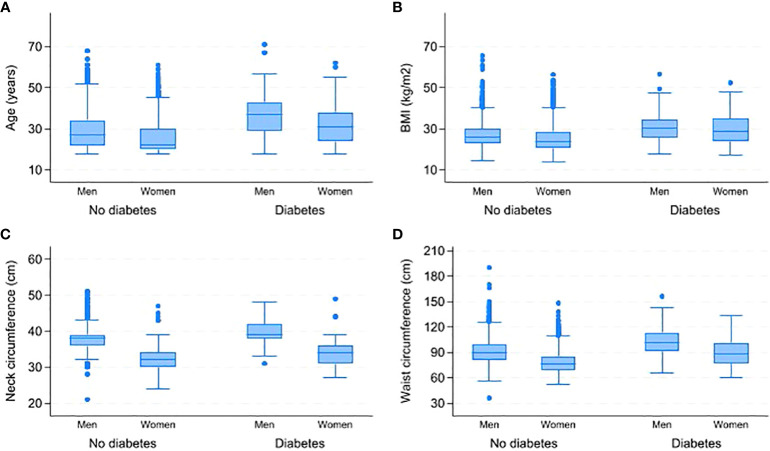
Comparison of age, BMI, neck and waist circumferences by gender and type 2 diabetes status. **(A)** Distribution of age by gender and diabetes status. **(B)** Distribution of BMI by gender and diabetes status. **(C)** Distribution of neck circumference by gender and diabetes status. **(D)** Distribution of waist circumference by gender and diabetes status. BMI-Body Mass Index.

**Table 3** displays the gender-based variations in responses to the STOP-BANG questionnaire. A total of 555 respondents (12.12%) reported snoring, with 15.03% of men and 8.45% of women reporting snoring. Over two-thirds of women (68.02%) reported feeling tired, while 48.96% of men reported tiredness. Men and women have similar rates of witnessed stop breathing while sleeping (11.66% and 11.22% respectively) and BMI ≥ 35 (9.51% of men and 9.44% of women). However, high blood pressure is more prevalent in men, with a rate of 29.24% compared to 11.62% in women. Only a small portion of the sample was above 50 years old (1.88%). **Figure 2** displays a box plot of age, BMI, waist, and neck circumference by gender and type 2 diabetes status. Men have higher median values for age, neck, and waist circumference than women. People with diabetes of both sexes have higher median age, BMI, and neck and waist circumference than non-diabetics. **Supplementary Data Figure S1** shows the distribution of age, BMI, neck and waist circumference by gender and OSA risk status. Men have higher neck and waist measurements across the three OSA risk groups. However, women had a higher median age and BMI in the high OSA risk group than men.

**Table 3:** Comparison between participant responses to the STOP-BANG questionnaire based on gender.

STOP-BANG Criteria	Male n=2555 (55.81%)	Female n=2023 (44.19%)	Total Sample n=4578
**Do you Snore?**	384 (15.03)	171 (8.45)	555 (12.12)
**Do you often feel Tired?**	1251 (48.96)	1376 (68.02)	2627 (57.38)
**Has anyone Observed you stop breathing?**	298 (11.66)	227 (11.22)	525 (11.66)
**Having High Blood Pressure**	747 (29.24)	235 (11.62)	982 (21.45)
**Having BMI≥35**	243 (9.51)	191 (9.44)	434 (9.48)
**Age>50**	53 (2.07)	33 (1.63)	86 (1.88)
**Neck circumference > 41cm for male and > 43 cm for female**	621 (24.31)	10 (0.49)	631 (13.78)

The data is presented as numbers (percentages) for the total sample of 4578 participants who answered yes for each STOP-BANG question.

BMI, body mass index.

Results of the unadjusted and adjusted logistic regression analysis are presented in **Table 4**. In the univariate analysis, a significant association was found between type 2 diabetes and intermediate OSA risk OR=2.44 (95% CI: 1.78-3.35; p-value <0.0001), high OSA risk OR=6.44 (95% CI: 4.32-9.59; p-value < 0.0001), education attainment of middle school or below OR=2.18 (95% CI: 1.30-3.66; p-value < 0.003), waist circumference above cut-off value OR=3.26 (95% CI: 2.44-4.37; p-value <0.0001), being married OR=2.71 (95% CI: 2.03-3.62; p-value <0.0001), or being a widow or divorced OR=2.98 (95% CI: 1.62-5.47; p-value <0.0001). Smoking was also found to have marginal significance with type 2 diabetes with OR=1.25 (95% CI: 1.00-1.55; p-value 0.041). In the multivariate analysis, factors remained significantly associated with type 2 diabetes were: Intermediate OSA risk OR=1.65 (95% CI: 1.18-2.32; p-value <0.004), high OSA risk OR=3.44 (95% CI: 2.23-5.33; p-value < 0.0001), waist circumference OR=2.16 (95% CI: 1.57-2.96; p-value <0.0001), and being married OR=**1.95** (95% CI: **1.43-2.65**; p-value <0.0001). Factors of education attainment of middle school or below and OR=1.73 (95% CI: 1.01-2.97; p-value < 0.045) or being a widow or divorced OR=2.24 (95% CI: 1.20-4.18; p-value < 0.011) were statistically significant in the multivariate analysis. Smoking and educational attainment of university or more were not statistically significant in the multivariate analysis. In **Table 4**, the multivariate logistic regression model showed better performance using AIC in predicting the association between OSA and type 2 diabetes than the univariate logistic regression model.

**Table 4:** Regression analysis of the association between OSA risk, predictors and type 2 diabetes.

	Univariate		Multivariate	
	OR (95%CI)	p-value	OR (95%CI)	P value
*Intermediate OSA risk*	*2.44 (1.78-3.35)*	*<0.0001*	*1.65 (1.18-2.32)*	*0.004*
*High OSA risk*	*6.44 (4.32-9.59)*	*<0.0001*	*3.44 (2.23-5.33)*	*<0.0001*
*Education-Middle school or less*	*2.18 (1.30-3.66)*	*0.003*	*1.73 (1.01-2.97)*	*0.045*
*Education-University or more*	*0.99 (0.74- 1.34)*	*0.981*	*0.92 (0.68-1.24)*	*0.582*
*Waist circumference*	*3.26 (2.44-4.37)*	*<0.0001*	*2.16 (1.57-2.96)*	*< 0.0001*
*Marital status-Married*	*2.71 (2.03-3.62)*	*<0.0001*	*1.95 (1.43-2.65)*	*< 0.0001*
*Marital status-Others*	*2.98 (1.62-5.47)*	*<0.0001*	*2.24 (1.20-4.18)*	*0.011*
*Smoking*	*1.25 (1.00-1.55)*	*0.041*	*1.01 (0.79-1.28)*	*0.968*
*χ ^2^ (df, p-value)	*80.33 (<0.0001)*		*136.60 (P value <0.0001)*	
*AIC (df)*	*1642.411 (3)*		*1600.608 (10)*	

Data is presented as odds ratio (95%, confidence intervals).

For the univariate and multivariate models, the reference groups were: "low-OSA risk" for OSA risk, "Secondary education" for educational attainment, "within average range" for categorical waist circumference, "single" for marital status and "No" for smoking.

Others in marital status include widows or divorced.

OSA, Obstructive sleep apnea; χ2, Likelihood Ratio Test chi-squared; df., degree of freedom; AIC, Akaike's information criterion.

*Univariate logistic regression analysis with type 2 diabetes as an outcome and OSA as a predictor.

## Discussion

In our study, the overall prevalence of intermediate OSA risk was 7.60%, while high OSA risk was 16.58% among Emirati adults. In contrast to our study, the reported prevalence of high OSA risk was higher in the studies conducted in Dubai and Kuwait, which reported a rate of high OSA risk of 21% and 20.0%, respectively (8, 9). The discrepancies in prevalence rates may be attributed to the distinct characteristics of the study population and sample size. Our study utilized a larger sample size (n=4578), and our participants were generally younger than those in the studies conducted in Dubai (n=1214) and Kuwait (n=651). The mean age of our participants was 27.5 years, while the mean age for Dubai and Kuwait study participants was 39.95 and 34.0 years, respectively (8, 9).

Our study used the STOP-BANG questionnaire to assess the risk of OSA. The significant relationship between OSA and type 2 diabetes found in our results is consistent with studies hat used polysomnography, the gold standard for OSA diagnosis (39–41). This alignment supports the validity of our findings despite the different diagnostic methods. While polysomnography provides more precise diagnoses, the STOP-BANG questionnaire is a validated and practical tool for large-scale screenings and has shown comparable effectiveness in identifying high-risk individuals (42). However, it is important to note that questionnaire-based screening may lead to underestimation or overestimation of OSA prevalence compared to polysomnography.

Our analysis showed that participants with diabetes have a threefold increased risk for high OSA risk. Yet, within the subset of patients with type 2 diabetes, the prevalence of high OSA risk was lower than the figures reported in the existing literature on type 2 diabetes. In our study, we found that 9.22% of diabetic participants had a high risk of OSA compared to non-diabetic. This finding was lower than the outcomes of a study conducted in Saudi Arabia, where 15.2% of type 2 diabetes patients showed a high risk of OSA (11). Additionally, a Chinese study found that the prevalence of OSA using polysomnography in type 2 diabetes patients was 17.5% (43). The discrepancies in the results can be explained by the difference in population characteristics, the lower overall prevalence of type 2 diabetes in our study population and the difference in OSA and type 2 assessment approaches.

We observed a statistically significant association between OSA risk and type 2 diabetes after adjusting for education attainment, waist circumference, marital status, and smoking (**Table 4**). Compared to individuals with a secondary level of education, a lower level of education (middle school or less) was significantly associated with diabetes in intermediate and high OSA risk groups (**Table 4**). In contrast to our results, a Jordanian study found no significant association between educational attainment and OSA risk in diabetes patients (44). Lower educational attainment may be associated with an unhealthy lifestyle characterized by more obesity and physical inactivity, which are risk factors for OSA and type 2 diabetes (45). Furthermore, educational level influences health literacy, which is critical for understanding and managing chronic conditions like OSA and type 2 diabetes (46). Individuals with lower educational levels may have limited knowledge about healthy eating, the importance of regular exercise, and effective diabetes and OSA management strategies (46–48). Therefore, addressing educational disparities could be a crucial strategy in reducing the prevalence of OSA and type 2 diabetes. Public health interventions that focus on improving health literacy, promoting healthy lifestyles, and increasing access to healthcare for individuals with lower educational attainment could help mitigate these risks.

In agreement with previous studies, we found that being married, divorced or widowed (others) was significantly associated with diabetes compared to being single in both adjusted and non-adjusted models (49, 50). The risk of intermediate and high OSA also remained significant with type 2 diabetes when adjusting for marital status (as shown in **Table 4**). This may be due to the fact that the married, divorced, or widowed groups are older than the single group. Older age is a known risk factor for OSA and diabetes as well (12, 21, 51). We also noted that the high OSA risk was more prevalent in the married group than in the single, divorced, or widowed groups, as shown in **Table 1**. This could be because married individuals are more likely to report symptoms of snoring and stopped breathing while asleep, as these symptoms are likely to be recognized and reported by their sleep partners (50). Moreover, previous research indicates that being married may lead to a higher reported prevalence of health issues such as OSA, possibly due to increased awareness and treatment-seeking behavior (50, 52). Additionally, the stress associated with major marital changes, like divorce or the loss of a spouse, may exacerbate chronic health conditions and potentially elevate the risk of developing type 2 diabetes (53). These findings highlight the complex relationship between marital status and the risk of OSA and type 2 diabetes, influenced by factors such as age, stress levels, and health behaviors (21, 45). Therefore, it is essential to consider these existing studies to gain a comprehensive understanding of the complex interplay between marital status and health outcomes.

The relationship between OSA and type 2 diabetes is multifaceted and involves several pathophysiological mechanisms. Research indicates that OSA-induced hypoxia and hypopnea attributed to low oxygen levels can impact the hypothalamic-pituitary-adrenal axis HPAA (54, 55). Activation of HPAA can trigger the release of stress markers and cause elevated cortisol levels, oxidative stress, and increased inflammatory stress markers (55, 56). The resulting increase in stress markers can lead to several harmful processes, including endothelial dysfunction, beta-cell dysfunction, insulin resistance, and abnormal glucose metabolism (14, 55). Habitual snoring and stopped breathing are linked to more abnormal glucose metabolism and insulin resistance in snorer individuals compared to non-snorers, which can exacerbate the progression of type 2 diabetes (14, 57). Similar to our results, studies have shown that men are more likely to report snoring, which may explain the higher prevalence of OSA among men (58).

In our sample, there was a linear increase in the prevalence of high OSA risk with obesity (see **Table 1**; **Supplementary Data Figure S1**). The prevalence of BMI ≥ 35 was higher in the high OSA risk group compared to the low OSA risk group (49.77% vs 28.80%). Furthermore, higher mean BMI was found in the diabetes group (30.31%) compared to non-diabetic group (26.31%), as presented in **Table 2**. Previous studies have revealed a positive correlation between a higher BMI and a greater risk of OSA in type 2 diabetes patients (6, 59). In our study, we found that men had higher waist and neck circumference compared to women, and waist circumference was significantly associated with the risk of OSA and type 2 diabetes.

Although the prevalence of intermediate and high OSA risk was higher in men than women, we noticed that women had a higher BMI than men in the intermediate and high OSA risk groups, as illustrated in **Supplementary Data Figure S1**. Moreover, the BMI was also higher in diabetic women compared to diabetic men (see **Figure 2**). These results match a study results that found women with type 2 diabetes have higher BMI compared to men (60). Our results suggest potential sex differences in the pathogenesis and health impact of OSA. Although the complete mechanism is not fully understood, evidence suggests that males typically have more central obesity and greater fat accumulation in the neck compared to females (19, 60). Central obesity, as waist circumference indicates, is related to metabolic and inflammatory changes and may play a more significant role in the development of OSA and T2DM in males than in females (14, 57, 61). Moreover, the accumulation of fat in the neck may contribute to airway blockage and subsequent breathing disturbances during sleep, which are more prominent in males (58). Additionally, hormonal differences, such as lower levels of progesterone in men, may contribute to reduced respiratory drive and increased susceptibility to OSA (62). More research is needed to explore the underlying mechanisms and potential therapeutic strategies for addressing sex-specific differences in the pathogenesis and health impact of OSA.

Although there is biological plausibility for the association between smoking and the risk of OSA in type 2 diabetes patients, our results do not provide adequate evidence to establish a significant relationship between smoking and this association. It is worth noting that the stigma attached to smoking may have led to underreporting of smoking, especially in women, which could have influenced the lack of correlation we observed (63). Our results are consistent with the findings of Amin et al., who also reported that current smoking did not affect the association between OSA and type 2 diabetes (64).

### Study strengths and limitations

Our study had strengths in different areas compared to other studies. In our study, diabetes was confirmed clinically using standard laboratory methods and classified according to the American Diabetes Association using standardized procedures to ensure high data quality (65). Furthermore, we used objective measurements to collect the anthropometric data using standardized procedures (e.g., waist, weight). Compared to the cross-sectional study conducted in Dubai, our study included a large sample size of Emirati nationals by using the UAHFS data, making our results more applicable to the Emirati population (8). Furthermore, our sample included mainly young adults and women who are often underrepresented in studies examining OSA and type 2 diabetes. In our analysis, we considered important confounding factors related to OSA. To our knowledge, there has been no prior research conducted on the relationship between OSA risk and type 2 diabetes, as well as the gender-based disparities concerning OSA risk symptoms experienced by Emirati participants.

There are several potential limitations to this study, and therefore, the results should be interpreted carefully. First, due to the nature of the study design, the causal relationship of the variable cannot be assessed, so results should be interpreted as associative rather than causal. Second, the risk of OSA was evaluated using self-report methods rather than polysomnography, the gold standard assessment tool for OSA (2). While self-reported methods are common in survey-based studies, they may introduce recall and misclassification bias, potentially leading to over- or underrepresentation of OSA risk (66). However, the STOP-BANG questionnaire, which we used, has demonstrated high sensitivity in detecting individuals at low, intermediate, and high risk for OSA (31). Furthermore, in our data, five of the eight criteria in the STOP-BANG questionnaire were measured using objective methods. The UAHFS does not collect data on diabetes type. However, we believe that the majority of diabetic subjects had type 2 diabetes based on their medication type and the onset of the disease. Despite adjusting for multiple factors, the influence of residual confounders, such as family history, physical activity, and alcohol consumption, cannot be entirely excluded. The final sample size was reduced due to high rates of missing data and uncertain responses, including “prefer not to answer” or “do not know” in the STOP-BANG survey.

### Sensitivity analysis

**Supplementary Table S1** (see supplementary data) shows that individuals categorized under intermediate OSA risk had a significantly higher likelihood of the outcome (type2 diabetes), with an odds ratio (OR) of 1.40 (95% CI: 1.02, 1.93, Z = 2.09, p = 0.036). Similarly, the high OSA risk category also showed a significant association with the outcome, with an OR of 1.40 (95% CI: 1.06, 1.85, Z = 2.36, p = 0.018). These findings suggest that specific OSA categories are important predictors of the outcome.

Waist circumference emerged as a significant predictor, with higher waist categories being strongly associated with the outcome (OR = 2.21, 95% CI: 1.78, 2.76, Z = 7.06, p < 0.0001). These findings highlight the significant impact of waist circumference on the outcome. Education level showed mixed results. The category of middle school or less was not significantly associated with the outcome (OR = 1.17, 95% CI: 0.86, 1.60, Z = 1.00, p = 0.317), while the education category of university or higher was significantly associated with a lower likelihood of the outcome (OR = 0.83, 95% CI: 0.69, 0.99, Z = -1.97, p = 0.049). Smoking status did not show a significant association with the outcome, with an OR of 1.00 (95% CI: 0.81, 1.24, Z = 0.02, p = 0.982), (see **Supplementary Data Table S1**). Marital status also played a crucial role; individuals classified as married had an OR of 2.36 (95% CI: 1.94, 2.88, Z = 8.53, p < 0.0001), and those under others (widows or divorced) had an OR of 3.27 (95% CI: 2.17, 4.92, Z = 5.69, p < 0.0001).

Overall, the sensitivity analysis confirms that certain factors, such as specific OSA categories, waist circumference, and marital status, are significantly associated with the outcome. In contrast, other variables, like low education levels and smoking status, show weaker or no significant associations. This analysis underscores the importance of considering multiple imputations to account for missing data and provides a robust check on the stability of our findings.

## Conclusions

Based on a large sample of Emirati participants, our study has revealed that individuals with a high risk of OSA are more likely to have type 2 diabetes as compared to those with a low risk of OSA. The study has also identified significant associations between the risk of OSA and type 2 diabetes with factors of educational level, marital status, and waist circumference. To determine the long-term and directional consequences of OSA on type 2 diabetes, further research is required by conducting longitudinal studies utilizing polysomnography, the standard diagnostic tool for OSA. Such research would help us comprehend the progression and potential causal relationships over time. In the meantime, our findings stress the significance of routine OSA screening in diabetic patients, given the high prevalence of OSA risk among this population. Early identification and management of OSA can improve glycemic control and overall health outcomes. The findings emphasize the need for integrated care approaches, tailored patient education, and lifestyle interventions, especially for high-risk groups and those with lower educational attainment. Addressing both OSA and diabetes through comprehensive, multidisciplinary strategies can enhance patient quality of life and reduce the burden of these chronic conditions. Healthcare providers can improve the overall management of type 2 diabetes and patient outcomes by proactively assessing and addressing OSA.

## Data availability statement

The data analyzed in this study is subject to the following licenses/restrictions: Data can be made available upon a reasonable request. Requests to access these datasets should be directed to mkt6@nyu.edu.

## Ethics statement

The studies involving humans were approved by the Institutional Review Board at New York University Abu Dhabi, Dubai Health Authority, Ministry of Health and Prevention in the UAE, and Health Research and Technology Committee, reference number DOH/HQD/2020/516, reference number DOH/HQD/2020/516. The studies were conducted in accordance with the local legislation and institutional requirements. Written informed consent for participation was not required from the participants or the participants’ legal guardians/next of kin because this study used previously collected data from the UAEHFS.

## Author contributions

MT: Conceptualization, Data curation, Formal analysis, Methodology, Project administration, Software, Visualization, Writing – original draft, Writing – review & editing. AAh: Formal analysis, Supervision, Writing – review & editing. MA: Writing – review & editing. AAl: Writing – review & editing. YI: Writing – review & editing. AAb: Writing – review & editing. RA: Funding acquisition, Supervision, Writing – review & editing.
