# Corrigendum: Association between obstructive sleep apnea risk and type 2 diabetes among Emirati adults: results from the UAE healthy future study

**DOI:** 10.3389/fendo.2024.1516500

**Published:** 2024-11-29

**Authors:** Manal Taimah, Amar Ahmad, Mohammad Al-Houqani, Abdulla Al Junaibi, Youssef Idaghdour, AbdiShakur Abdulle, Raghib Ali

**Affiliations:** ^1^ Public Health Research Center, New York University Abu Dhabi, Abu Dhabi, United Arab Emirates; ^2^ Department of Medicine, College of Medicine and Health Sciences, UAE University, Al-Ain, United Arab Emirates; ^3^ Department of Pediatrics, Zayed Military Hospital, Abu Dhabi, United Arab Emirates; ^4^ MRC Epidemiology Unit, University of Cambridge, Cambridge, United Kingdom

**Keywords:** obstructive sleep apnea, type 2 diabetes, gender differences, obesity, UAEHFS, United Arab Emirates

In the published article, there was an error in the title of **Table 1** as published. The authors forgot to add the word “risk”. The corrected legend appears below.

“Table 1 General characteristics of the study participants by obstructive sleep apnea risk severity.”

In the published article, there was an error in **Table 1** as published. The value for “Intermediate OSA risk” in the second row, column 3, was incorrectly reported as “Intermediate OSA risk 887 (7.60%)”. The correct text is “Intermediate OSA risk 348 (7.60%)”.

The corrected **Table 1** and its caption appear below.

**Table 1:** General characteristics of the study participants by obstructive sleep apnea risk severity.

Characteristics	Low OSA risk 3471 (75.82%)	Intermediate OSA risk 348 (7.60%)	High OSA risk 759 (16.58%)	P Values
Age, mean (±SD)	26.25 (7.51)	30.97 (9.37)	34.17 (9.51)	<0.0001

Gender, n (%)				<0.0001
Male	1604 (62.78)	275 (10.76)	676 (26.46)	
Female	1867 (92.29)	73 (3.61)	83 (4.10)	

Marital status, n (%)				<0.0001
Single	2,366 (83.11)	158 (5.55)	323 (11.35)	
Married	997 (63.46)	171 (10.88)	403 (25.65)	
Others	108 (67.50)	19 (11.88)	33 (20.62)	

Education attainment, n (%)				<0.0001
Middle school or less	122 (59.22)	30 (14.56)	54 (26.21)	
Secondary school	1514 (76.62)	151 (7.64)	311 (15.74)	
University or more	1726 (76.61)	157 (6.97)	370 (16.42)	
Missing PN, DN	109 (76.22)	10 (6.99)	24 (16.78)	

BMI, mean (±SD)	24.97 (5.15)	29.91 (6.62)	36.74 (6.97)	<0.0001
BMI, n (%)				<0.0001
≤24.9	1882 (90.70)	46 (2.22)	147 (7.08)	
25-29.9	1060 (76.15)	88 (6.32)	244 (17.53)	
30-34.9	404 (59.68)	121 (17.87)	152 (22.45)	
≥35	125 (28.80)	93 (21.43)	216 (49.77)	

Neck circumferences, mean (±SD)	34.28 (3.60)	38.76 (3.67)	42.39 (3.07)	<0.0001
Neck circumference, n (%)				
Below cut-off value	3356 (85.03)	104 (2.63)	487 (12.34)	<0.0001
Above cut-off value	115 (18.23)	244 (38.67)	272 (43.11)	

Waist circumference, mean(±SD)	81.40 (12.71)	96.33 (14.66)	112.83 (15.16)	<0.0001
Waist circumference, n (%)				<0.0001
Below cut-off value	2400 (86.18)	81 (2.91)	304 (10.92)	
Above cut-off value	1071 (59.73)	267 (14.89)	455 (25.38)	

Diabetic status, n (%)				< 0.0001
No	3362 (77.0)	315 (7.21)	689 (15.78)	
Yes	109 (51.42)	33 (15.57)	70 (33.02)	

Having High blood pressure, n (%)				<0.0001
No	3132 (87.10)	201 (5.59)	263 (7.31)	
Yes	339 (34.52)	147 (14.97)	496 (50.51)	

Smoking, n (%)				<0.0001
No	2519 (82.83)	182 (5.98)	340 (11.18)	
Yes	761 (60.02)	136 (10.73)	371 (29.26)	
Missing, PN, DN	191 (71.00)	30 (11.15)	48 (17.84)	

The total sample (n=4578) values are presented as numbers (%) or mean (±SD). Continuous variables by OSA status, were tested using ANOVA, and a Chi-squared test was used for categorical variables. Missing data represents "Prefer not to answer" or "Do not know" or missing. If data was missing, a missing indicator was added to the table. OSA, obstructive sleep apnea; SD, standard deviation; BMI, body mass index; PN, prefer not to answer; DN, do not now.

The authors apologize for these errors and state that this does not change the scientific conclusions of the article in any way. The original article has been updated.

