# Supplementary material for: Association between obstructive sleep apnea risk and type 2 diabetes among Emirati adults: results from the UAE healthy future study
Source: Front Endocrinol (Lausanne). 2024 Jul 16;15:1395886. doi: 10.3389/fendo.2024.1395886 (PMC11286458; doi:10.3389/fendo.2024.1395886)
Supplement: Supplementary file 1 [file DataSheet_1.zip › Sensitivity_analysis_June.docx]

**Association between obstructive sleep apnea risk and type 2 diabetes among Emirati adults: Results from the UAE Healthy Future Study**

Manal Taimah^1*^, Amar Ahmad^1^, Mohammed Al-Houqani^2^, Abdulla Al Junaibi^3^, Youssef Idaghdour^1^, AbdiShakur Abdulle^1^, Raghib Ali^1&4^

^1^ Public Health Research Center, New York University Abu Dhabi, Abu Dhabi, United Arab Emirates

^2^ Department of Medicine, College of Medicine and Health Sciences, UAE University, Al-Ain, United Arab Emirates

^3^ Department of Pediatrics, Zayed Military Hospital, Abu Dhabi, United Arab Emirates

^4^ MRC Epidemiology Unit, University of Cambridge, Cambridge CB2 1TN, UK

**Correspondence**

Manal Taimah, Public Health Research Center, New York University Abu Dhabi, Abu Dhabi P.O. Box 129188, United Arab Emirates. Phone: +971 02 628 4824. E-mail address: mkt6@nyu.edu

| **Table S1** Results of the sensitivity analysis from a logistic regression using 5 multiple imputations with type 2 diabetes as an outcome | | | |
| --- | --- | --- | --- |
|  | OR (95% CI) | z-value | p-value |
| Intermediate OSA risk | 1.402 (1.022, 1.925) | 2.092 | 0.036 |
| High OSA risk | 1.398 (1.059, 1.846) | 2.365 | 0.018 |
| Education-Middle school or less | 1.172 (0.859, 1.599) | 1.000 | 0.317 |
| Education-University or more | 0.830 (0.690, 0.999) | -1.972 | 0.049 |
| Waist circumference | 2.213 (1.775, 2.760) | 7.059 | < 0.0001 |
| Marital status-Married | 2.363 (1.939, 2.879) | 8.534 | < 0.0001 |
| Marital status-Others | 3.268 (2.173, 4.916) | 5.685 | < 0.0001 |
| Smoking | 1.002 (0.812, 1.237) | 0.023 | 0.982 |
| Data is presented as odds ratio (95%, confidence intervals).  For the multivariate models, the reference groups were: "low-OSA risk" for OSA risk, "Secondary education" for educational attainment, "Within average range" for categorical waist circumference, "single" for marital status and "No" for smoking.  Others in marital status include widows or divorced.  Abbreviations: OSA, Obstructive sleep apnea | | | |

The results indicate that individuals at intermediate and high risk for OSA have a significantly higher likelihood of the outcome (type 2 diabetes). Higher waist circumference is strongly associated with the outcome, while higher education levels are associated with a lower likelihood. Smoking status shows no significant association. Marital status significantly impacts the outcome, with married individuals and those who are widowed or divorced showing higher odds. These findings highlight the importance of OSA risk categories, waist circumference, and marital status in predicting the outcome.
