# Supplementary material for: Association between obstructive sleep apnea risk and type 2 diabetes among Emirati adults: results from the UAE healthy future study
Source: Front Endocrinol (Lausanne). 2024 Jul 16;15:1395886. doi: 10.3389/fendo.2024.1395886 (PMC11286458; doi:10.3389/fendo.2024.1395886)

**Association between obstructive sleep apnea risk and type 2 diabetes among Emirati adults: Results from the UAE Healthy Future Study**

Manal Taimah^1*^, Amar Ahmad^1^, Mohammed Al-Houqani^2^, Abdulla Al Junaibi^3^, Youssef Idaghdour^1^, AbdiShakur Abdulle^1^, Raghib Ali^1&4^

^1^ Public Health Research Center, New York University Abu Dhabi, Abu Dhabi, United Arab Emirates

^2^ Department of Medicine, College of Medicine and Health Sciences, UAE University, Al-Ain, United Arab Emirates

^3^ Department of Pediatrics, Zayed Military Hospital, Abu Dhabi, United Arab Emirates

^4^ MRC Epidemiology Unit, University of Cambridge, Cambridge CB2 1TN, UK

**Correspondence**

Manal Taimah, Public Health Research Center, New York University Abu Dhabi, Abu Dhabi P.O. Box 129188, United Arab Emirates. Phone: +971 02 628 4824. E-mail address: mkt6@nyu.edu


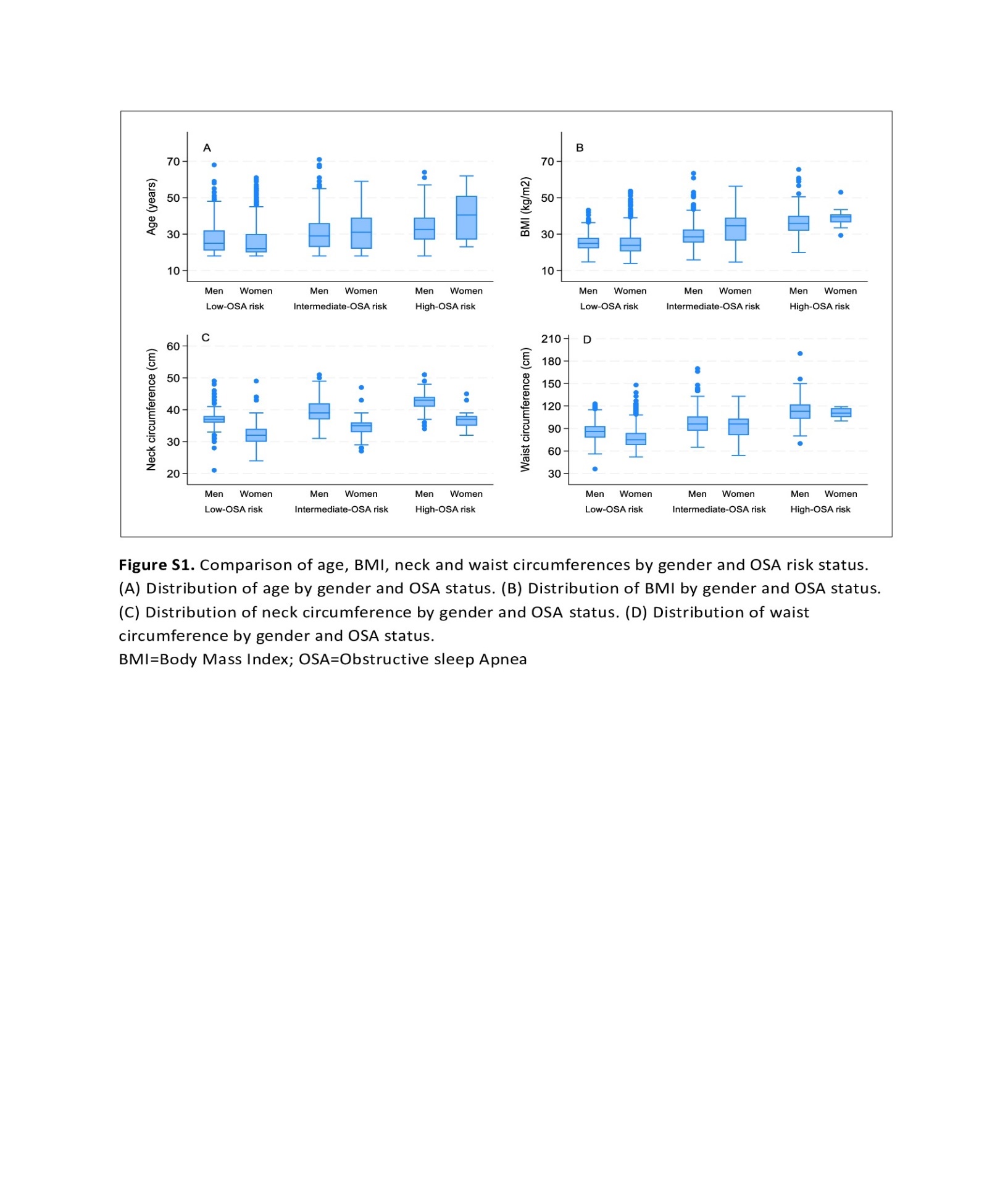

Supplement: Supplementary file 1 [file DataSheet_1.zip › Supplementary_figure_S1.docx]
